# Characterization and In Vitro and In Vivo Evaluation of Tacrolimus-Loaded Poly(ε-Caprolactone) Nanocapsules for the Management of Atopic Dermatitis

**DOI:** 10.3390/pharmaceutics13122013

**Published:** 2021-11-26

**Authors:** Guilherme dos Anjos Camargo, Leandro Ferreira, Diego José Schebelski, Amanda Martinez Lyra, Fernanda Malaquias Barboza, Bruna Carletto, Adriana Yuriko Koga, Betina Christi Semianko, Daniele Toniolo Dias, Leandro Cavalcante Lipinski, Andressa Novatski, Vijayasankar Raman, Jane Manfron, Jessica Mendes Nadal, Paulo Vitor Farago

**Affiliations:** 1 Postgraduate Program in Pharmaceutical Sciences, Department of Pharmaceutical Sciences, State University of Ponta Grossa, Ponta Grossa 84030-900, Brazil; guuicamargo.gc@gmail.com (G.d.A.C.); leandro-ferreira20@hotmail.com (L.F.); diego.ski@hotmail.com (D.J.S.); amandinhamlyra@gmail.com (A.M.L.); fer_barboza@hotmail.com (F.M.B.); brunacarletto@hotmail.com (B.C.); adri_yuriko@hotmail.com (A.Y.K.); janemanfron@hotmail.com (J.M.); jessicabem@hotmail.com (J.M.N.); 2 Academic Department of Physics, Federal University of Technology-Paraná-UTFPR, Ponta Grossa 84017-220, Brazil; betachristi@gmail.com (B.C.S.); danieletoniolodias@gmail.com (D.T.D.); 3 Post-Graduate Program of Health Sciences, State University of Ponta Grossa, Ponta Grossa 84030-900, Brazil; leandrolipinski@yahoo.com.br (L.C.L.); anovatski2@gmail.com (A.N.); 4 Department of Physics, State University of Ponta Grossa, Ponta Grossa 84030-900, Brazil; 5 National Center for Natural Products Research, School of Pharmacy, University of Mississippi, Oxford, MS 38677, USA; vraman@olemiss.edu

**Keywords:** anti-inflammatory activity, autoimmune skin disease, drug release, FK506, immunomodulator, macrolide lactone

## Abstract

Background: Tacrolimus (TAC) is a drug of natural origin used in conventional topical dosage forms to control atopic dermatitis. However, direct application of the drug often causes adverse side effects in some patients. Hence, drug nanoencapsulation could be used as an improved novel therapy to mitigate the adverse effects and enhance bioavailability of the drug. Methods: Physicochemical properties, in vitro drug release experiments, and in vivo anti-inflammatory activity studies were performed. Results: TAC-loaded nanocapsules were successfully prepared by the interfacial deposition of preformed polymer using poly(ε-caprolactone) (PCL). The nanoparticulate systems presented a spherical shape with a smooth and regular surface, adequate diameter (226 to 250 nm), polydispersity index below 0.3, and suitable electrical stability (−38 to −42 mV). X-ray diffraction confirmed that the encapsulation method provided mainly the drug molecular dispersion in the nanocapsule oily core. Fourier-transform infrared spectra suggested that nanoencapsulation did not result in chemical bonds between drug and polymer. In vitro drug dissolution experiments showed a controlled release with a slight initial burst. The release kinetics showed zero-order kinetics. As per the Korsmeyer–Peppas model, anomalous transport features were observed. TAC-loaded PCL nanocapsules exhibited excellent anti-inflammatory activity when compared to the free drug. Conclusions: TAC-loaded PCL nanocapsules can be suitably used as a novel nano-based dosage form to control atopic dermatitis.

## 1. Introduction

Atopic dermatitis (AD), or eczema, is a dermatological, inflammatory, non-infectious, and chronic disease characterized by long-lasting itching of the affected skin and involves a combination of genetic factors that affect the skin barrier and the immune system [1]. The disease affects about 25% of children and 1 to 3% of adults worldwide [2,3,4]. The common symptoms include skin rashes, erythema, papules, localized exudative lesions, and dry skin. The condition can affect sleep and daily activities leading to discomfort and poor quality of life [1]. The physiopathology of AD is complex, multifactorial, and difficult to understand due to the number of factors that influence it, such as genetic diseases, defects in the epidermal barrier, unsettled immune system, and disturbed skin microbiota [1].

According to Wollenberg et al. (2021) [2], several treatments for AD and patients with transient levels are available. The most common remedies include avoiding the triggers that induce the disease, using emollients that improve epidermal function, nonspecific anti-inflammatory drugs, and therapies with immunosuppressants such as class II glucocorticoids and calcineurin inhibitors. For patients with moderate or recurrent AD, topical tacrolimus (TAC) or class II/III topical glucocorticoids combined with emollients and medical follow-up are recommended. Severe and persistent AD requires treatments with systemic immunosuppressants such as cyclosporin A.

TAC, also known as FK506 (molecular formula: C_44_H_69_NO_12_), is a hydrophobic macrolide lactone belonging to the class of calcineurin inhibitors, produced by the fermentation of *Streptomyces tsukubaensis* bacteria, a strain isolated from a soil sample collected near Mount Tsukuba. It was discovered in April 1983 by Fujisawa in his research laboratory with other natural products, mainly of microbial origin. TAC, like cyclosporine, inhibits calcineurin, leading to suppression of T cell activation. However, TAC has an immunosuppressive effect 10 to 100 times more potent than cyclosporine, with reduced vasoconstrictor and fibrinogenic effects. Its systemic use is widely utilized to prevent rejection prophylaxis after solid organ transplantation and its topical use is mainly for cutaneous autoimmune diseases, such as AD [5,6,7,8,9].

Physicochemically, TAC shows a high molecular mass of 822 Da, pKa 1 = 2.94; pKa 2 = 9.95; pKa 3 = 14.07; log P of 3.96 ± 0.83; and melting point in the range 126–129 °C. It is insoluble in water (4–12 µg/mL), slightly soluble in saturated hydrocarbons, and highly soluble in lipids and organic solvents such as methanol, ethanol, acetone, and propylene glycol (PPG). TAC is available as white crystals and sold as capsules, injectables, and ointments for topical application [10,11,12,13,14].

TAC has been approved by the Food and Drug Administration (FDA), and it is used in dermatological ointments at concentrations from 0.03% for children aged 2 to 16 years and at 0.1% for other patients. Due to a more selective mechanism of action that does not alter collagen synthesis, the topical calcineurin inhibitors can be used as corticosteroid-sparing agents as they are not associated with skin agenesis [7,8]. In general, topical TAC preparations were well tolerated by patients with autoimmune skin diseases, but significant adverse effects such as itching, burning, and a hot sensation in the application site at the start of treatment were reported [8,15]. In spite of corticotherapy, TAC is not associated with skin atrophy, stretch marks, or telangiectasia and may be more appropriate for use on skin areas more susceptible to these effects. Even though this is an advantage of using TAC, the adverse reactions affect patients’ adherence to treatment, leading to suspension of drug therapy [8].

Recent studies based on nanotechnology and human skin have shown that this novel delivery approach demonstrates greater drug deposition in the dermal layer and deeper penetration, increasing its therapeutic action and reducing adverse effects. Therefore, it is clinically recommended to formulate a drug delivery system to enhance drug permeation into the skin and thus reduce its topically applied dose for minimizing adverse effects and for maintaining efficacy at the same time. This delivery system is expected to have the ability to deposit more of the drug, persist in the skin longer, and not cause damage [12,13,16].

In recent years, polymeric nanocapsules (NCs) have attracted greater interest in drug delivery due to the structure of the encapsulated core. Compared to nanospheres, the oil core can effectively increase the drug encapsulation while reducing the polymer matrix content of the nanoparticles. Furthermore, the polymeric shell can isolate the encapsulated drug from the tissue environment, thus avoiding degradation or early release induced by pH, temperature, enzymes, and other biological factors. In addition, the shell can be functionalized by intelligent molecules capable of interacting with target biomolecules, allowing the delivery of drugs with higher accuracy. These advantages are the reasons for the great interest and the enhanced application of nanocapsules in the pharmaceutical field as a carrier of drugs and cosmetic actives [17]. Therefore, this study aimed to prepare, characterize, and evaluate the in vitro drug release and in vivo anti-inflammatory activity of TAC-loaded nanocapsules to be used as a novel alternative to conventional therapy for AD.

## 2. Materials and Methods

### 2.1. Reagents

Tacrolimus 99.3% pure (Mw = 822.0389 g/mol, Fagron, Rotterdam, Netherlands), poly(ε-caprolactone) (PCL) (Mw = 14,000 g/mol, Sigma-Aldrich, St. Louis, MO, USA), sorbitan monooleate (Span^®^ 80, Oxiteno, Mauá, Brazil), polysorbate 80 (Tween^®^ 80, Delaware, Porto Alegre, Brazil), medium chain triglycerides (MCT, 99% pure, Focus Química, São Paulo, Brazil), lactose monohydrate (LAC, Biotec Produtos Químicos, São José dos Pinhais, Brazil), and acetone (≥99.9% pure, Vetec Química, Rio de Janeiro, Brazil) were used as received. Water was purified in a Milli-Q Plus water purification system (Millipore, Bedford, MA, USA).

### 2.2. Preparation of Polymeric Nanocapsules Containing Tacrolimus (TAC)

**Figure 1:**
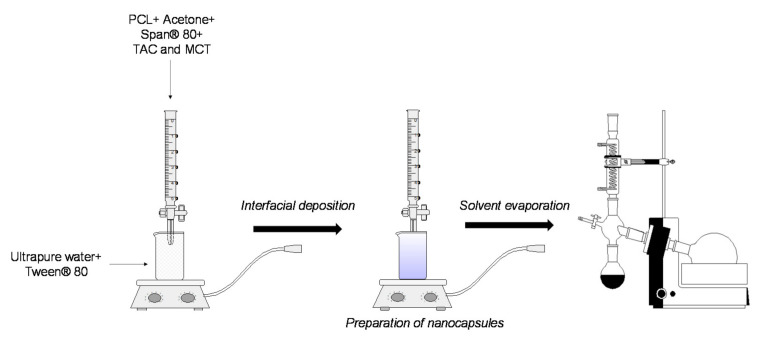
Scheme of the obtaining method of TAC-loaded PCL nanocapsules by interfacial deposition of preformed polymer.

TAC-loaded poly(ε-caprolactone) nanocapsule suspensions were prepared by interfacial deposition of preformed polymer method as described by Fessi et al. (1989) [18]. Briefly, PCL (0.100 g) was solvated in acetone (27 mL) in the presence of Span^®^ 80 (0.0770 g), TAC (0.010 g), and MCT (0.300 g) until solubilization under mechanical stirring at 3.500 rev/min and 40 °C (Fisaton equipment, 713 model, São Paulo, Brazil). The organic phase was then dripped onto the aqueous phase (53 mL) containing Tween^®^ 80 (0.0770 g). The mixture was kept under mechanical stirring at 3.500 rev/min at 40 °C for 10 min. The organic solvent was evaporated under reduced pressure in a rotary evaporator (Tecnal, TE-211 model, Piracicaba, Brazil) to the final volume of 10 mL for obtaining the loaded formulation at 1 mg/mL (NC-1) of TAC. The experiment was carried out from six different batches. A suspension of nanocapsules with no drug (NC-N) was prepared as the negative control. Figure 1 is a schematic of the method used to prepare PCL nanocapsules containing or not TAC.

### 2.3. Characterization of Polymeric NCs Containing TAC

#### 2.3.1. Determination of Mean Diameter, Polydispersity Index (PDI), and Zeta Potential of NCs

Mean diameter, PDI, and zeta potential were measured (*n* = 3) by photon correlation spectroscopy (PCS) according to dynamic light scattering (DLS) after diluting an aliquot of the NCs in ultrapurified water (1:500) (Zetasizer Nanoseries, Malvern Instruments, Malvern, UK). A one-way analysis of variance (ANOVA) was performed to verify the statistical difference between the mean diameters.

#### 2.3.2. Field Emission Scanning Electron Microscopy (FESEM) and Transmission Electron Microscopy (TEM)

The PCL NCs formulations were lyophilized with LAC monohydrate as a cryoprotectant agent. LAC was dispersed in the NCs formulations under magnetic stirring. Then, the suspension was frozen in a ULT upright freezer (Thermo Scientific, model ULT-2186-3-D37, Waltham, MA, USA) at −80 °C for 24 h and freeze-dried in a lyophilizer (LIOTOP, L202, São Paulo, Brazil) for 48 h until complete removal of water.

The freeze-dried samples were sputter-coated with gold using an IC-50 ion coater metallizer (SHIMADZU, Kyoto, Japan). Morphological evaluation of the NCs was carried out and images were prepared using a FESEM (TESCAN, model Mira 3, Brno, Czech Republic) at an acceleration voltage of 8 to 10 kV.

Then, 5 µL of the nanosuspensions were placed on a transmission electron microscope (TEM) grid and dried at room temperature (25 ± 2 °C). The morphology of the NCs were examined using TEM equipment (JEM 1200 EX-II, JEOL, Tokyo, Japan).

#### 2.3.3. X-ray Diffraction (XRD)

Pure TAC, PCL, physical mixture (PM), and NCs were examined by XRD in a Shimadzu XRD-6000 diffractometer (Kyoto, Japan), 2°/min and 2θ scan from 5° to 60°, copper Kα radiation (λ = 1.5418 Å), current of 40 mA, and voltage of 40 kV for the observation of possible peaks indicative of crystallinity.

#### 2.3.4. Fourier-Transform Infrared Spectroscopy (FTIR)

The formulations were analyzed by FTIR using potassium bromide (KBr) pellets, using 4 mg of each sample and 196 mg of spectroscopic grade KBr (2%, m/m), in the IR Prestige-21 equipment (Shimadzu, Kyoto, Japan), in the range of 4000–500 cm^−1^, with a resolution of 2 cm^−1^ and 64 scan/min. The spectra obtained were evaluated against the spectra of the pure TAC, PCL, PM, LAC, and NC-N.

### 2.4. In Vitro Drug Dissolution Study

In vitro release studies were carried out using vertical Franz diffusion [19,20] with a 12 mL receiving compartment and an available area for diffusion of 1.76 cm^2^. Dialysis cellulose membranes (molecular weight cut-off (MWCO) 12–14 kDa; pore size 2.4 nm, D9652, Sigma-Aldrich, St. Louis, MO, USA), which were previously hydrated in 70% ethanol [21] for 24 h, were used for the experiments.

Subsequently, these membranes were placed between the donor and receiver compartments of vertical cells. In the donor compartment, 1 mL of the solution containing each sample to be tested (TAC in PPG or NC-1) was placed at a concentration equivalent to 0.1% *w*/*v* of TAC. The nanocapsule suspension (formulation NC-1) was used immediately after its preparation and did not require any resuspension procedure. The receptor medium was entirely filled with 70% ethanol (12 mL) for ensuring the sink condition. The system was kept under constant stirring at 32 ± 2 °C and occluded to avoid evaporation of the samples.

Next, 2 mL aliquots were removed from the receptor phase at previously determined time intervals. The same volume was returned to the system. The study was conducted in sextuplicate.

The samples obtained were analyzed by ultra-high-performance liquid chromatographic–photodiode array (UHPLC-PDA) using the previously developed and validated method [22]. The drug mass accumulated in the receptor compartment at each time was calculated, considering the total cell volume, the total amount of drug removed in each collection, and the effective area of release in the cell.

To determine the NC release kinetics, the in vitro results were fitted to the mathematical models of zero order, first order, Higuchi, Korsmeyer–Peppas, and Hixson–Crowell. The fitting of the experimental data to the model was performed by linearization and the adjusted R-squared was used as model selection criteria.

### 2.5. Evaluation of In Vivo Anti-Inflammatory Activity

This experimental protocol was previously approved by the Committee of Animal Use and Ethics of the State University of Ponta Grossa under registration number 0069285/2019.

#### 2.5.1. Animals

Fifty male albino mice (*Mus musculus* species, Balb/c strain) (25–35 g) aged approximately 90 days were used in this study. The animals were kept at the Advanced Centre for Life Studies at the State University of Ponta Grossa at 22 ± 2 °C in an automatically controlled 12 h-light/dark cycle with free access to water and feed.

The animals were taken to the experimental laboratory at least one hour before the beginning of the experiments. The tests were carried out with 5 experimental groups, divided according to Table 1. As per the previously performed statistical planning, 10 animals per group were used.

#### 2.5.2. Induction of Atopic Dermatitis in BALB/c Mice

AD was induced by topical application of 12-*O*-tetradecanoylphorbol-13-acetate (TPA, 2.5 μg) in DMSO (20 μL) to the right ear of mice. TAC was solubilized in PPG at 0.1% (*w*/*v*). TAC solution, non-loaded, and TAC-loaded nanocapsule suspensions were recently prepared and then administered subcutaneously in the dorsal region of the animal neck six hours after the treatment with TPA [23]. TAC was used as a positive control (G4). The inflammatory control group (ICG) received only TPA (G2).

After 24 h, 2 mice per group were sacrificed and 6 mm circles of mouse ear tissue were collected. The experiment took place for 10 days, interspersing treatment and euthanasia days.

#### 2.5.3. Fibrinogen Dosage

The animals were anesthetized with isoflurane and blood was then collected by cardiac puncture.

Fibrinogen dosage was performed according to Shalm, Jain, and Carroll (1975) [24] using the plasma precipitation technique at 56 °C. In summary, an aliquot of the blood sample was centrifuged in a capillary tube, and the serum refractive index was measured. Another aliquot of the blood was heated at 56 °C for 5 min and centrifuged, and the refractive index was also measured. Fibrinogen concentration was calculated by subtracting the values obtained before and after fibrinogen precipitation.

#### 2.5.4. Histological Analysis

Ear samples collected from mice were sent to the Curitiba Technical Histopathological Center for the preparation of histological slides stained with hematoxylin and eosin (HE) [25]. The histological sections with the inflammatory infiltrate and the thickness of the epidermis were photographed in an Olympus AX70 optical microscope (magnification of 20 and 40×) (Waltham, MA, USA) using the T capture software version 5.1.1 (Tucsen, Fuzhou, China). The quantification of inflammatory cells was performed using the ImageJ software—cell counter version 1.44 (U.S. National Institutes of Health, Bethesda, MD, USA), counting the entire field of view.

### 2.6. Statistical Analysis

Results are presented as mean ± standard error of the mean. Statistical significance between groups was assessed by ANOVA, followed by Tukey’s post-hoc test. The significance level accepted for the tests was *p* < 0.05.

All analyses were performed using GraphPad Prism version 6 statistical software (San Diego, CA, USA).

## 3. Results and Discussion

### 3.1. Preparation of NCs

The TAC-loaded and non-loaded PCL nanocapsules (NC-1 and NC-N) were successfully obtained by the interfacial deposition of preformed polymer method. All formulations showed a liquid aspect with a slightly bluish-white opalescent color as previously reported [26]. The encapsulation efficiency of TAC-loaded PCL nanocapsules was 99.39% [22]. This formulation (NC-1) was stable for 60 days [27].

### 3.2. Characterization of NCs Containing Tacrolimus

#### 3.2.1. Determination of Mean Diameter, Polydispersity Index (PDI), and Zeta Potential of NCs

Both formulations showed a mean diameter of 226.64 nm (NC-1) and 250.53 nm (NC-N). The statistical analysis showed no significant difference between these nanoformulations, indicating that the drug did not have a detrimental effect in size. Table 2 summarizes the results obtained.

According to Schaffazick et al. (2003) [28], polymeric nanoparticles generally have a diameter range of 100–300 nm. This feature depends on various factors such as the formulation, preparation method, nature of the oil used in the NC core, polymer characteristics, and the presence/absence of the drug.

Regarding the PDI, all formulations presented values below 0.3, indicating a homogeneous size distribution of the NCs and a unimodal behavior. A uniform particle size distribution is obtained when the organic and aqueous phases are mixed quickly to form a homogeneous dispersion [28,29,30].

The zeta potential demonstrated a suitable electrostatic stability of the particles, as it reflects the surface potential, and its value must be greater than ±30 mV for representing an appropriate superficial charge. This adequate stability is due to large repulsive forces that prevent particles from aggregating in case of occasional collisions [28,31].

Thus, the particle size values and the surface properties may play an important role in NC bioactivity by influencing the in vitro drug release. The smaller particle size leads to a larger surface area, providing a rapid release from these nanosystems. In addition, they may enhance the interaction with bacterial cells, cellular uptake, cytotoxicity of these nanomaterials, and their pharmacokinetics by increasing the therapeutic efficacy of the drug encapsulated [31,32].

#### 3.2.2. Field Emission Scanning Electron Microscopy (FESEM) and Transmission Electron Microscopy (TEM)

**Figure 2:**
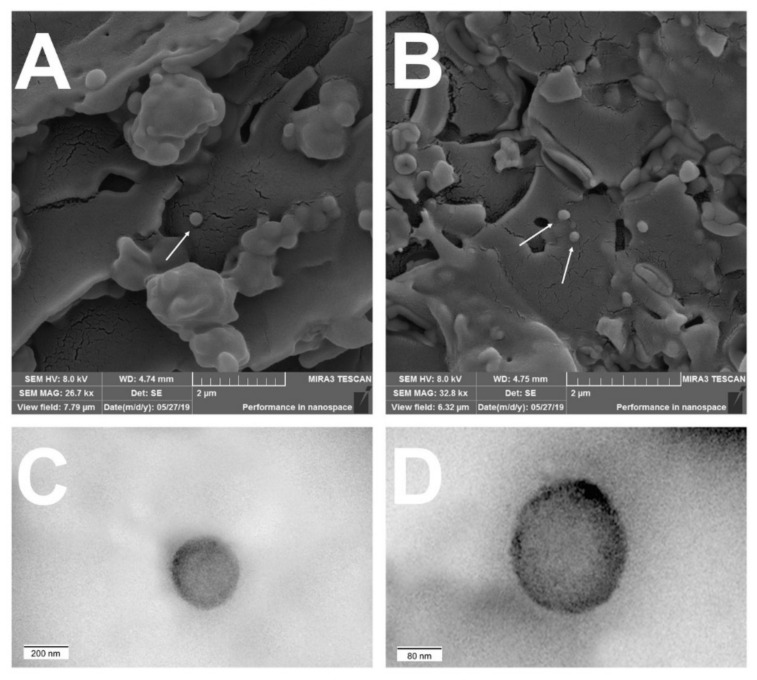
FESEM images of non-loaded and TAC-loaded nanocapsules: (**A**) NC-N at 26,700× magnification and (**B**) NC-1 at 32,800× magnification. TEM images of non-loaded and TAC-loaded nanocapsules: (**C**) NC-N at 25,000× magnification and (**D**) NC-1 at 62,500× magnification.

Figure 2 depicts the photomicrographies obtained by FESEM and TEM of the NC formulations. It was possible to observe particles with a well-defined spherical shape and a smooth and uniform surface by FESEM. TEM image (Figure 2C) confirmed the structure of shell-oily core nanocapsule. Figure 2D confirmed the smooth surface and the absence of pores. These results are consistent with the literature in which NCs were also prepared from PCL and by the chosen method [32,33,34,35,36,37,38]. In addition, no presence of drug crystal was reported on particle surface.

#### 3.2.3. X-ray Diffraction (XRD)

**Figure 3:**
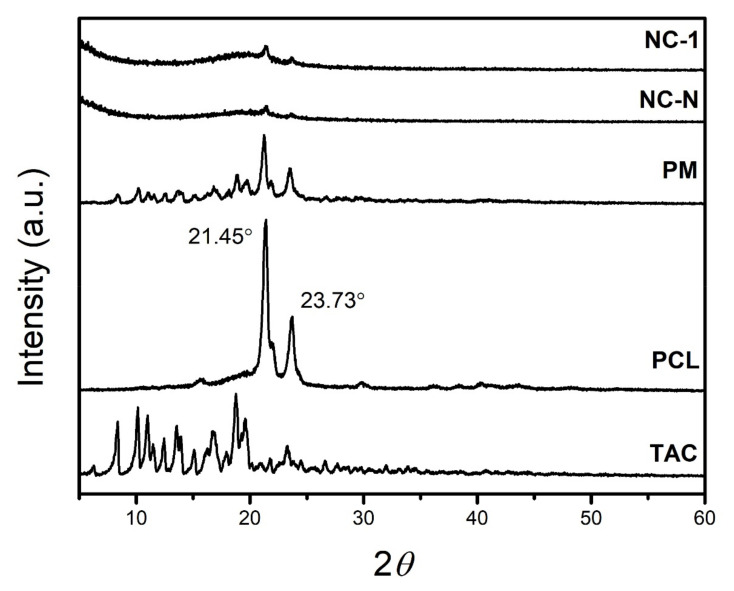
Difractograms of TAC, PCL, PM, and non-loaded (NC-N) and TAC-loaded (NC-1) nanocapsules obtained by XRD analysis.

The XRD data for TAC, PCL, PM, non-loaded, and TAC-loaded nanocapsules are shown in Figure 3. Regarding the pure drug, intense peaks of crystallinity between 2θ = 5 and 25° were achieved as previously reported [39,40,41,42,43], while PCL proved its semi-crystalline nature with characteristic peaks at 2θ of 21.45 and 23.73° [30,44,45]. The PM shows crystallinity peaks coincident with those observed for the drug and the polymer. The mild mixture using mortar and pestle does not change the crystallinity of either TAC or PCL [46].

Considering the NC formulations, no drug crystallinity peak was seen, which indicates that TAC amorphization occurred during the interfacial deposition of preformed polymer method. In all formulations, the crystallinity peaks of PCL appeared.

Dantas et al. (2018), Obaidat et al. (2017), and Wang et al. (2016) [39,41,43] observed that the TAC incorporation into nanosystems leads to a disorder in the crystalline system and thus reduces its structural organization. The absence of drug peaks in the XRD of NCs may be due to the low amount of the drug or the total conversion of the drug into its amorphous form during their preparation. Moreover, Serrano et al. (2015) [47] reported that particle size reduction leads to a decrease in peak intensity, and its broadening can make it more difficult to accurately quantify the drug crystallinity in nanoparticles.

#### 3.2.4. Fourier-Transform Infrared Spectroscopy (FTIR)

FTIR analysis was performed to assess the presence of characteristic functional groups in TAC-loaded PCL nanocapsules, as well as possible changes in the spectrum due to molecular interactions between the drug and the other formulation components [48,49].

**Figure 4:**
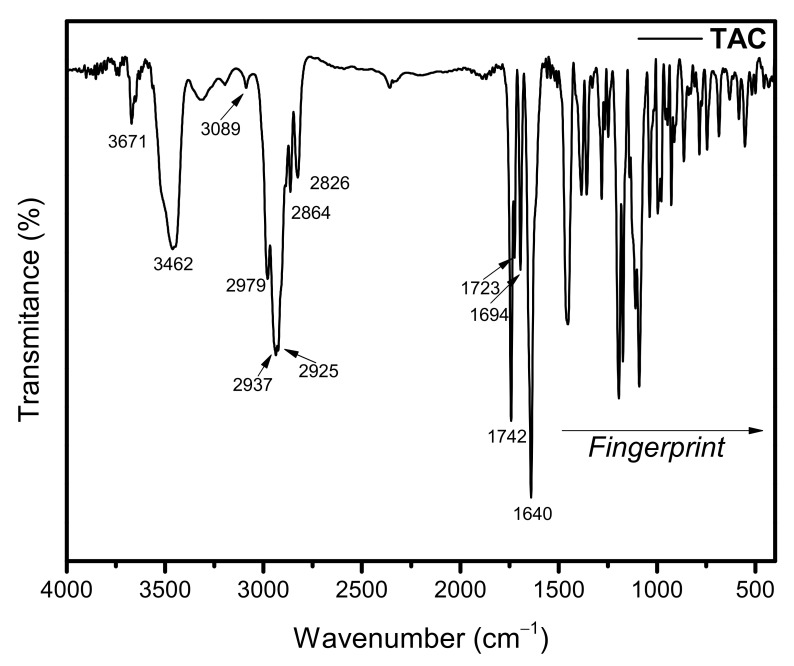
FTIR spectrum of TAC.

Figure 4 shows the FTIR spectrum of pure TAC, which showed a band of free OH stretch vibration at 3671 cm^−1^ and a band of OH in intermolecular hydrogen bond at 3462 cm^−1^. Stretch vibrations of sp^2^ hybridized carbon at 3089 cm^−1^ and of sp^3^ hybridized carbon at 2979, 2937, 2925, and 2864 cm^−1^ were also assigned. O–CH_3_ stretch vibration at 2826 cm^−1^, C=O stretch vibration of ester at 1742 cm^−1^, C=O stretch vibrations of ketone at 1723 and 1694 cm^−1^, and C=O/C=C stretch vibrations of amide at 1640 cm^−1^ were recorded. Other signals below 1500 cm^−1^ were due to the angular vibrations and the fingerprint of drug molecule. Signs similar to those assigned were also found in the literature [40,41,50,51,52].

**Figure 5:**
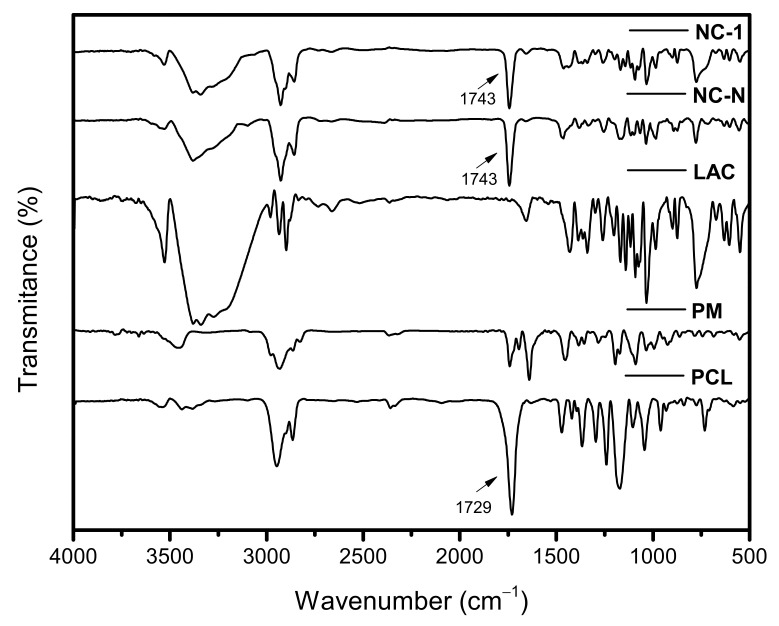
FTIR spectra of PCL, physical mixture, lactose, non-loaded, and TAC-loaded nanocapsules.

The other FTIR spectra of PCL, PM, LAC monohydrate, and non-loaded and TAC-loaded NCs are detailed in Figure 5. The FTIR spectrum of PCL showed typical bands of the polymer as stretch vibrations of sp^3^ hybridized carbon at 2948, 2899, and 2865 cm^−1^, C=O stretch vibration of ester at 1729 cm^−1^, angular vibrations of sp^3^ hybridized carbon at 1473 cm^−1^, C–O symmetric stretch vibrations at 1297 and 1241 cm^−1^, C–O asymmetric stretch at 1175 cm^−1^, and angular vibrations of sp^3^ hybridized carbon at 731 cm^−1^. The absorption bands attributed to the PM corresponded to the superposition of the FTIR spectra of the pure drug and the polymer without any change.

LAC monohydrate had a band of OH stretch vibration from intermolecular hydrogen bond at 3530 cm^−1^ and a broadening OH signal at 3300 cm^−1^. sp^3^ hybridized carbon stretch vibrations at 2980, 2934, 2898, and 2879 cm^−1^, C–O stretch vibration/asymmetric C–O–C stretch of ether at 1035 cm^−1^, and OH angular vibration at 775 cm^−1^ were assigned.

Regarding the spectra of non-loaded and TAC-loaded NCs, signals referring to the LAC used for lyophilization and bands belonging to the PCL, mainly due to C=O stretch vibration of the polyester at 1743 cm^−1^, were confirmed. The slight shift in the PCL C=O stretching from pure polymer (1729 cm^−1^) was probably related to the NC preparation method. No FTIR signals of TAC were recorded for NC-1 spectrum, which may suggest the drug encapsulation.

Hence, it is concluded that the characteristic bands of the drug have disappeared. There was no evidence of considerable displacement of the main bands when compared with those in the spectra of the isolated substances, which represented that no remarkable physicochemical interaction between the components of the formulation occurred. Thus, these results may indicate that the TAC can be entirely encapsulated and that the polymer is only serving as a drug carrier.

### 3.3. In Vitro Drug Dissolution Study

**Figure 6:**
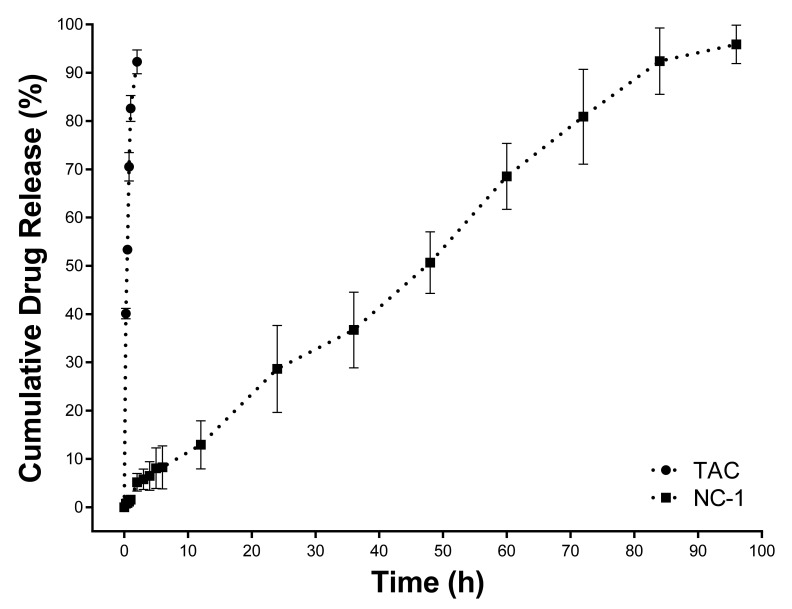
In vitro TAC release profile for free drug and TAC-loaded nanocapsules (NC-1).

In vitro release assay was performed with free TAC and NCs containing the drug. The values obtained for these two samples from six different batches at each collection time interval were corrected considering the removed aliquots. Figure 6 shows the mean release profiles.

Pure TAC showed a rapid release profile and reached 80% of drug release in 60 min of the dissolution experiment. Regarding NC-1, a drug-controlled release was achieved with a slight burst effect (12.93% within 720 min (12 h)), reaching 80% of TAC release after 4320 min (72 h).

The drug dissolution test is widely used to investigate the drug release from a pharmaceutical dosage form to change the drug pharmacokinetics and eventually improve its pharmacological effect. Drug release studies are essential to know the behavior of different pharmaceutical dosage forms. They are widely used for the development and optimization of all types of sustained, delayed, or controlled release systems. This experiment is essential for nanosystems that use polymers or lipids for transporting drugs to the desired sites [53,54].

In addition to the drug release assay, it is possible to know the release kinetics by using mathematical models. These models are based on phenomena such as dissolution, diffusion, swelling, erosion, precipitation, and/or degradation. The drug release kinetics are driven by mechanisms that depend on the matrix composition, geometry, preparation method, and dissolution medium used for the drug release. They also depend on the main factors affecting the release, such as the mean particle diameter, physical state, the drug concentration within polymer particles, the viscoelastic properties of the polymer system, and the dissolution-diffusion properties of the encapsulated drug. Regarding the release by the polymer, there are two main release mechanisms: drug diffusion through the polymer matrix and polymer erosion [53].

Therefore, several kinetic models have been used for fitting in vitro release data based on the experimental results found. These results can be fitted to models such as zero-order, first-order, Higuchi, Hixson–Crowell, Korsemeyer–Peppas, and others [53,54].

According to the kinetic models applied to the release profiles (Table 3), it was possible to demonstrate that NC-1 showed a zero-order release (r^2^ adjusted = 0.9947). The zero-order model refers to the process of constant drug release by a concentration-independent drug delivery system. This model is ideal for a controlled release dosage form with minimal fluctuations in drug levels in tissue or circulation [53,54]. Pharmaceutical dosage forms that present this behavior release the same amount of drug per unit of time and are one of the best approaches to formulate drugs for prolonged release [55].

The biodegradability of polymers is an attribute explored in the modulation of drug release. Due to the presence of ester groups in its chemical structure, PCL is susceptible to hydrolysis. Therefore, it can undergo chemical or enzymatic hydrolysis, and the polymer degradation mechanism indirectly alters this release. In PCL formulations, non-enzymatic cleavage begins in the amorphous region, which is self-catalyzed by the carbonyl end groups of the fragmented polymer chain. The water permeability in the formulation is the limiting step for this fragmentation and thus producing smaller fragments depending on the molecular weight of the PCL. This mass process is usually accompanied by surface erosion caused by grooves and cracks in the surface [56,57]. Thus, it is feasible to hypothesize that hydrolytic changes in the PCL shell of NCs may have influenced the drug release process to the dissolution medium.

In order to elucidate the drug release mechanism from PCL NCs, the Korsmeyer–Peppas model can be used, which is indicated when the release mechanism is not well known or when more than one type of release mechanism may be involved. In this formula, the n value reports the type of physical phenomenon associated with the release. For delivery systems with spherical geometry, an n value less than 0.45 indicates that the release is controlled by diffusion (Fickian diffusion); an n value above 0.89 represents the drug release by polymer erosion (case II transport or non-Fickian transport mechanism); and an n value between 0.45 and 0.89 characterizes the anomalous transport with features of both mechanisms [53,54,55,58]. Taking all these into account, the formulation NC-1 showed an n value of 0.8816 by the Korsmeyer–Peppas model, which indicated that the physical mechanism of drug release from the TAC-loaded PCL occurred by the anomalous transport, in which the drug was released by the both Fickian diffusion and relaxation/polyester erosion as aforementioned [53].

### 3.4. Evaluation of In Vivo Anti-Inflammatory Activity

#### 3.4.1. Fibrinogen Dosage

Fibrinogen is a plasma glycoprotein with a wide range of activities in the hemostasis system. This protein is a product of three genes, *FGA*, *FGB*, and *FGG*, and it is mainly synthesized by hepatocytes, although extrahepatic fibrinogen synthesis is observed in the lung, the kidney, and other tissues. Fibrinogen is an acute-phase protein because its plasma concentration increases quickly in the presence of inflammatory processes. In adequate concentrations, this protein supports controlled inflammation, even allowing the synthesis of other proteins, such as collagen in the dermis. Hepatic fibrinogen synthesis can increase 20-fold from baseline levels under conditions of severe stress [59,60].

**Figure 7:**
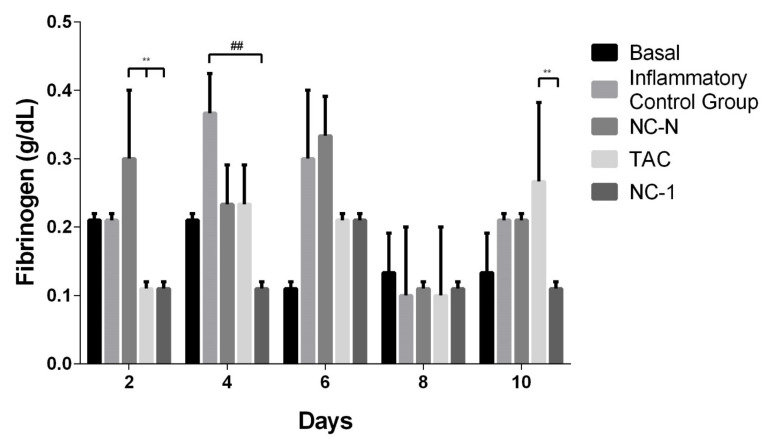
Effect of TAC, non-loaded, and TAC-loaded nanocapsules (NC-N and NC-1) on serum fibrinogen in TPA-induced male Balb/c mice. Statistical analysis performed on each group on days 2, 4, 6, 8, and 10 of treatment. ANOVA statistical test with Tukey post-test, with significance of **/## *p* < 0.01, when compared with the inflammatory control group (ICG) or between TAC, NC-N, and NC-1.

Figure 7 represents the quantification of fibrinogen for the studied groups during the 10 days of treatment. NC-1 maintained low values for fibrinogen in all time intervals tested. NC-1 provided a statistically lower fibrinogen value than pure TAC on the tenth day. In that sense, TAC-loaded NCs demonstrated better inflammation control than the other treatment groups.

Acute inflammation is known to shift the homeostatic balance towards a prothrombotic and antifibrinolytic state in which there is an increase in the circulation of these mediators. The potency of fibrinogen as an inflammatory mediator is linked to the ability to influence multiple leukocyte aspects through direct and indirect mechanisms. Fibrinogen can facilitate the transmigration of leukocytes out of the capillaries and induce effector functions of these cells, serving as a local, spatially defined clue within the damaged tissue [61].

In the case of neutrophils, fibrinogen performs this function by modulating the generation of second messengers, the production of reactive oxygen species, and the cell adhesion under inflammatory conditions. These findings demonstrate that the leukocyte–fibrinogen interaction can alter leukocyte function and therefore lead to changes in cell migration to the affected tissue [62].

Thus, the complexity of fibrinogen as an inflammatory mediator is dictated by the specific location or interactions in the microenvironment and the involvement of specific binding partners, making it clear that the reduction in circulating fibrinogen demonstrates a reduction and control of inflammation and, consequently, tissue damage [61]. Thus, it was possible to conclude that TAC-loaded nanocapsules had a better result in reducing acute inflammation when compared to other treatment groups.

#### 3.4.2. Histological Analysis

Repeated application of 12-*O*-tetradecanoylphorbol-13-acetate (TPA) in mice ears causes allergic dermatitis, resulting in infiltration of inflammatory cells such as lymphocytes, eosinophils, and mast cells [23]. In order to investigate whether TAC and the NC-1 formulation influenced the infiltration of inflammatory cells into the skin after TPA application, tissue sections were stained with HE to reveal the cells and layers of the ears.

**Figure 8:**
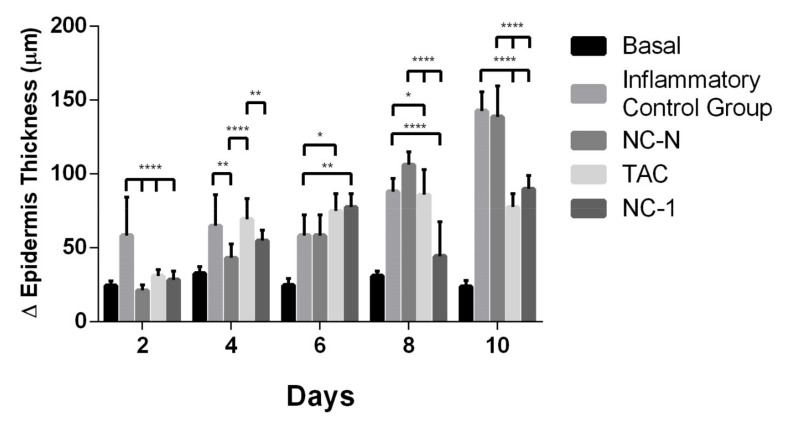
Effect of TAC, non-loaded, and TAC-loaded nanocapsules (NC-N and NC-1) on the variation of epidermis thickness of male Balb/c mice ears induced by TPA. Statistical analysis performed on each group on days 2, 4, 6, 8, and 10 of treatment. ANOVA statistical test with Tukey post-test, with significance of * *p* < 0.05; ** *p* < 0.01; and **** *p* < 0.0001.

Figure 8 depicts the variation on epidermis thickness of the different groups at treatment time intervals. TAC and NC-1 presented statistically lower changes in epidermis thickness than the inflammatory control group on days 2, 8, and 10 of treatment, even with the chemical aggression of TPA. NC-1 group had a significant reduction in the epidermis thickness than TAC on days 4 and 8, suggesting a better inflammatory modulation from NC formulation than the pure drug. NC-0 also provided a significant reduction in the epidermis thickness than the inflammatory control group on days 2 and 4.

**Figure 9:**
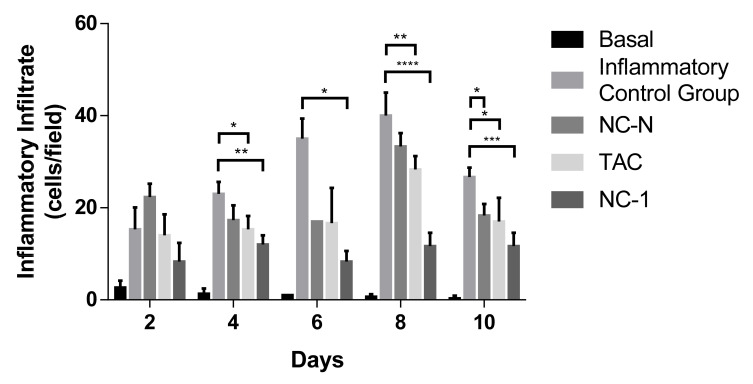
Effect of TAC, non-loaded, and TAC-loaded nanocapsules (NC-N and NC-1) on TPA-induced ear cell infiltration of male Balb/c mice. Statistical analysis performed on each group on days 2, 4, 6, 8, and 10 of treatment. ANOVA statistical test with Tukey post-test, with significance of * *p* < 0.05; ** *p* < 0.01; *** *p* < 0.001, and **** *p* < 0.0001, when compared to the inflammatory control group (ICG).

Regarding the inflammatory infiltrate (Figure 9), the migration of inflammatory cells to the ear dermal region in the groups that received TPA is indicative of the dermatitis induction. TAC and NC-1 significantly suppressed the cellular infiltration on days 4, 8, and 10 of treatment when compared with the inflammatory control group (ICG). NC-1 also revealed a significant statistical reduction in inflammation than ICG on day 6. Therefore, NC-1 has a relatively better performance than the free drug by providing an anti-inflammatory effect, possibly due to the controlled drug delivery.

**Figure 10:**
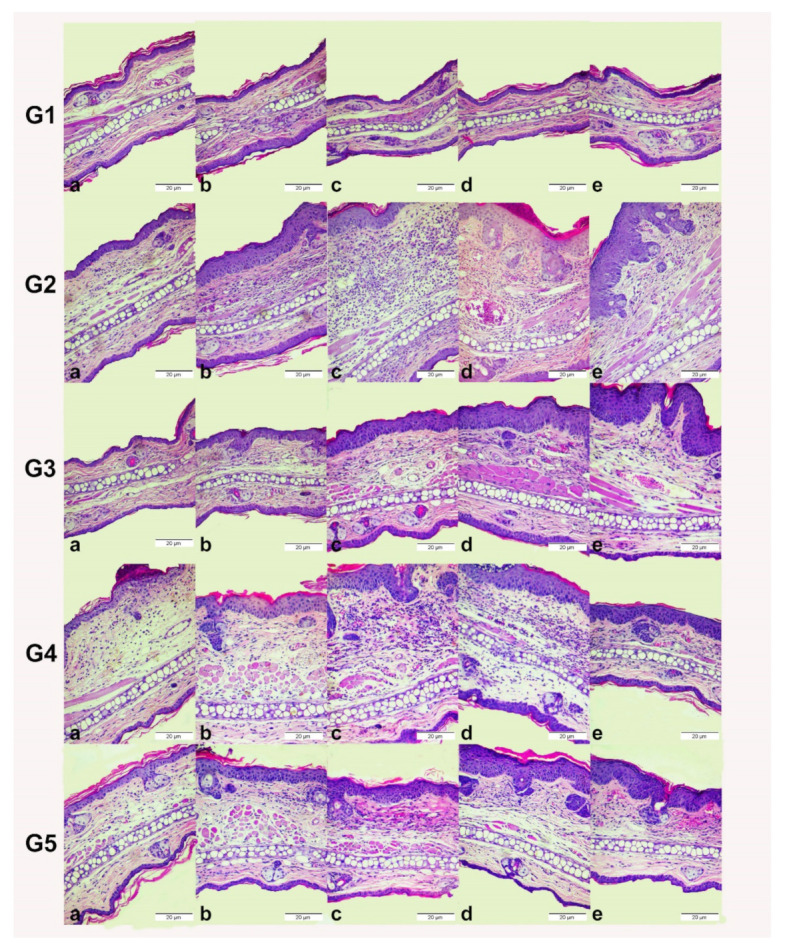
Effect of TAC, non-loaded, and TAC-loaded nanocapsules (NC-N and NC-1) on TPA-induced ear of male Balb/c mice. (**G1**) Basal group, (**G2**) inflammatory control group, (**G3**) NC-N group, (**G4**) TAC group, (**G5**) NC-1 group. (**a**) day 2, (**b**) day 4, (**c**) day 6, (**d**) day 8, and (**e**) day 10 of treatment. Images are representative of transverse sections of mice ears stained with hematoxylin and eosin (magnification: 200×; scale bar: 20 µm).

Figure 10 summarizes the histological sections of the male Balb/c mice ears from the different experimental groups on days 2, 4, 6, 8, and 10 of treatment. These results are consistent with the statistical analysis of the variation in epidermis thickness and of the inflammatory infiltrate. The basal group showed no ear edema. The inflammatory control group (ICG) revealed intense inflammation and hyperproliferative response to TPA consistent with the AD. Formulation NC-1 provided better control of the vasodilation, the polymorphonuclear leukocyte infiltration to the epidermis tissue, and the edema formation than NC-N and TAC groups. TAC group reduced the inflammatory histological features on the second and the tenth day of treatment.

Therefore, the histopathological results support the idea that NC-1 led to the in vivo effect of TAC with a higher suppression of T cell activation and a reduction of the inflammatory infiltrate. TAC-loaded PCL nanocapsules may also be a feasible alternative to the free drug by reducing the adverse effects of TAC and by improving the treatment adherence by patients [23].

The irritating agent TPA is a potent inflammatory and tumor-causing agent that leads to inflammation, intense cell hyperproliferation (T cells, neutrophils, and dendritic cells), erythema, and epidermal alterations, such as acanthosis [63,64]. Repeated topical application of TPA induces dermatitis in the ears of BALB/c mice, resulting in the immune response of T lymphocytes and a delayed strong allergic reaction that affects the thickness of their ear [65]. According to Boscardin (2012) [64], the cutaneous inflammatory response caused by this agent occurs by vasodilation and erythema development, followed by an increase in ear thickness due to cell extravasation. In addition, the adherence of polymorphonuclear leukocytes to the vessel wall and the degranulation of mast cells are observed.

The results reported for the inflammatory control group (ICG) represent the typical features of dermatitis, such as the prominent ear swelling and accumulation of inflammatory cells [65]. In the TPA-induced model used, the skin is thickened due to the hypertrophy and hyperkeratosis of epidermal cells and the increased concentration of inflammatory cells [12]. Thus, the allergic reaction caused by TPA is a useful model to assess the pharmacological effects of TAC-loaded PCL NCs. The dose chosen for the in vivo anti-inflammatory activity was in accordance with the conventional topical dosage forms at 0.1% [12,23,65].

Studies indicate two possible mechanisms that may underlie AD, which is inhibited by TAC-loaded PCL nanocapsules. The first was associated with the T cell type 2 (Th2) phenotype, and the second with the accumulation of large amounts of eosinophils, mast cells, and dendritic cells observed in the epidermis and dermis of patients with cutaneous autoimmune diseases [65]. Kandikattu and Mishra (2018) [66] reported that TAC, in addition to suppressing the Th1/Th2 cell activation, also suppresses the mast cell degranulation, reduces the primary sensory neurotransmitter release, causes the TRPV1 receptor desensitization, limits the IL-31 and IL-33 production, decreases the ST2 receptor mRNA expression, minimizes the impairment of TLR2/TLR1 balance, limits the periostin production, and reduces the pruritus and the scratching sensation in AD.

Other studies described in the literature developed different controlled release systems for TAC. In brief, these studies demonstrated a reduction of inflammation in cutaneous autoimmune diseases, such as AD and psoriasis. Gabriel et al. (2016) [63] used a hydrogel containing TAC for the control of psoriasis and observed a decrease in skin erythema, scaling, and thickness, indicating the treatment efficacy when compared to the topical ointment. Jain et al. (2019) [67] evaluated the effect of lipid and liposome-based nanoformulations against psoriasis and AD and verified a higher efficacy and safety profile. Yu et al. (2018) [12] prepared chitosan nanoparticles containing TAC, which were superior to conventional ointment for treating AD. However, none of them involved the preparation of polymeric NCs and their in vitro and in vivo evaluation for the management of AD.

The controlled release of TAC in nano-based systems directed to the desired site is mandatory to lead to a better treatment of cutaneous autoimmune diseases, such as dermatitis, psoriasis, vitiligo, among others, where the epidermal and dermal barrier is thickened due to hyperproliferation of keratinocytes and the formation of a plaque, resulting in a greater barrier to drug penetration [65]. The pharmacological results from the present paper demonstrate that the behavior of TAC was significantly improved using PCL nanocapsules as a novel controlled delivery system.

This remarkable improvement in the pharmacodynamic profile was achieved by a new nanocarrier that does not require any physical enhancement such as occlusion or ultrasound. This approach may lead to greater patient compliance, mainly when treating larger body regions. The combination of these in vivo results with suitable in vitro dissolution evidences the greater efficacy of the proposed nanoparticulate system when compared to the free drug.

## 4. Conclusions

In the present study, TAC-loaded polymeric nanocapsules were successfully obtained by the chosen method. The nanocapsules had a spherical shape with a smooth surface by FESEM and TEM. An adequate mean size for permeation through the skin layers and a suitable charge stability were achieved. The X-ray diffraction patterns and FESEM images confirmed the drug amorphization. FTIR analysis established that no chemical reaction occurred among the NC components. Drug dissolution experiments showed a controlled drug release with a slight initial burst. The release kinetics was better fitted to the zero-order equation. The Korsmeyer–Peppas model demonstrated a release mechanism based on the anomalous transport.

The in vivo anti-inflammatory activity of TAC-loaded nanocapsules revealed a better formulation effect when compared to the pure drug with a decrease in the epidermis thickness in Balb/c mice ears and a reduction in the inflammatory infiltrate. Histological analysis confirmed these findings. TAC-loaded PCL nanocapsules substantially prevented TPA-induced hyperplasia in the epidermis and avoided the migration of inflammatory cells. These results play an important role in dermatitis by inhibiting the immediate and chronic allergic reactions, resulting in improved clinical results and better patient compliance.

In summary, the TAC-loaded PCL nanocapsules may be used as a novel therapeutic alternative to the conventional treatment of atopic dermatitis with greater comfort and fewer adverse effects to the patient. The current evidence suggests that this nano-sized formulation improves the TAC effect and has additional benefits over the traditional ointment. These advantages are the reasons for the great interest and the enhanced application of nanocapsules in the pharmaceutical field as a carrier of drugs and cosmetic actives.

## Figures and Tables

**Table 1:** Group treatment scheme.

Group	Treatment
G1 (*n* = 10)	Animals that did not receive any treatment (basal group)
G2 (*n* = 10)	Animals treated with TPA (inflammatory control group—ICG)
G3 (*n* = 10)	Animals treated with TPA and NC-N (0.44 mL/animal ^1,3^) (NC-N Group)
G4 (*n* = 10)	Animals treated with TPA and TAC at a concentration of 0.1% (*w*/*v*) (0.4 mL/animal ^1^) (TAC Group)
G5 (*n* = 10)	Animals treated with TPA and TAC-loaded PCL nanocapsules at a drug concentration of 0.1% (*w*/*v*) (0.44 mL/animal ^1,2,3^) (NC-1 Group)

^1^ These volumes were firstly based on the mean weight of each mice group. ^2^ The drug encapsulation efficiency was also considered for TAC-loaded PCL nanocapsule suspensions. ^3^ The same volume was used for TAC-loaded and non-loaded PCL nanocapsules (NC-1 and NC-N).

**Table 2:** Mean diameter, polydispersity index (PDI), and zeta potential of the TAC-loaded (NC-1) and non-loaded (NC-N) nanocapsules.

Formulation	Mean Particle Size (nm)		PDI		Zeta Potential (mV)	
	Mean	SD *	Mean	SD *	Mean	SD *
NC-1	226.64	±32.28	0.23	±0.04	−38.11	±3.12
NC-N	250.53	±43.11	0.24	±0.04	−42.60	±5.58

* SD—standard deviation.

**Table 3:** Fitting data for in vitro TAC release profile from TAC-loaded nanocapsules (NC-1).

Model	Equation	Linear Equation	r^2^ Adjusted
Zero-order	*Q* = *Q*_0_ + *K*_0_*t*	y = 1.0506x + 1.511	0.9947
First-order	*dC/dT* = −*Kt*	y = −0.0122x + 2.0517	0.9143
Higuchi	*Q* = *KH t*^1/2^	y = 0.0945x + 1.2057	0.9532
Korsmeyer–Peppas	*Mt/M*∞ = *Kt^n^*	y = 0.8816x + 0.2487	0.9846
Hixson–Crowell	*Q*_0_^1/3^ − *Q*_1_^1/3^ = *KHC t*	y = −0.0294x + 4.7112	0.9675

Zero order: *Q* is the amount of drug released or dissolved, *Q*_0_ is the initial amount of drug in solution (it is usually zero), *t* is time, *K*_0_ is the zero order release constant; First order: *dC* is the concentration derivative, *dT* is the time derivative, *K* is the first order rate constant expressed in units of time^−1^; Higuchi: *KH* is the Higuchi dissolution constant; Kors–Peppas: *Mt*/*Mα* is the fraction of drug released at time *t*, *K* is the rate constant (having units of *t^n^*) incorporating structural and geometric characteristics of the delivery system. *n* is the release exponent indicative of the mechanism of transport of drug through the polymer; Hixson–C: *Qt*, denotes the remaining weight of solid at time *t*, *Q*_0_ is the initial weight of solid at time *t* = 0, and *KHC* represents the dissolution rate constant.
